# Influence of GPs’ unhealthy lifestyle on patients’ adherence to lifestyle recommendations: a cross-sectional study in Belgian primary care

**DOI:** 10.3399/BJGPO.2024.0221

**Published:** 2025-09-10

**Authors:** Julie Kerremans, Birgitte Schoenmakers

**Affiliations:** 1 Academic Center for General Practice, Department of Public Health and Primary Care, KU Leuven, Leuven, Belgium

**Keywords:** general practitioners, lifestyle, patient adherence, patient compliance, primary care

## Abstract

**Background:**

Research has shown that a physician’s lifestyle significantly affects patient adherence to lifestyle advice.

**Aim:**

To investigate the influence of GPs’ unhealthy lifestyles on patients’ adherence to lifestyle recommendations.

**Design & setting:**

A cross-sectional study was conducted from 19 April 2023–12 September 2023. Adults living in Flanders or Brussels with sufficient knowledge of Dutch were included.

**Method:**

Through a survey we studied the degree of willingness to follow lifestyle advice from GPs with unhealthy lifestyles. Secondary outcomes included the need for specific lifestyle advice, the feeling of being judged, and its impact on adherence.

**Results:**

Patients were less likely to follow lifestyle advice on smoking cessation (62.3%), alcohol use (64.9%), vaccination (49.7%), and eating habits (51.2%) from GPs exhibiting unhealthy behaviours in these areas. A significant portion (68.8%) indicated they were less likely to follow advice when feeling judged. Older responders were less likely to report being influenced by unhealthy GP habits, both in terms of adherence and perceived judgment (odds ratios [OR] 0.973 to 0.979). Similarly, responders with higher subjective physical health were less likely to be influenced by a physically inactive GP when receiving advice on physical activity (OR 0.799).

**Conclusion:**

The lifestyle of GPs appears to affect patients’ adherence to lifestyle advice, with unhealthy behaviours leading to decreased patient trust and adherence. Emphasising the importance of GPs maintaining a healthy lifestyle could enhance patient outcomes in lifestyle-related chronic disease prevention and management.

## How this fits in

This study contributes to the existing literature by highlighting the impact of GPs’ personal health behaviours on patient adherence to lifestyle recommendations. The lifestyle of GPs appears to affect patients’ adherence to lifestyle advice, with unhealthy behaviours leading to decreased patient trust and adherence. These insights are crucial for developing strategies to improve patient outcomes in lifestyle-related chronic disease prevention and management.

## Introduction

Global ageing brings many challenges. The population is not ageing in a healthy manner but rather with a high prevalence of chronic conditions. A healthy lifestyle is an important preventive and therapeutic measure for these conditions.^1–3^ Informing and helping to concretely implement health advice in daily life is part of the physician’s duties. This includes addressing smoking behaviour, alcohol use, eating habits, physical activity, sleep hygiene and rest, and stress and relaxation.^4,5^

Nearly all physicians acknowledge it as their task to provide lifestyle advice, with many recognising that their advice would be more effective if they themselves lead by example.^6^ Moreover, physicians with healthy eating habits and sufficient physical activity are more likely to advise on these topics, whereas physicians who smoke are less likely to discuss the smoking behaviour of their patients.^6–13^ Studies suggest that physicians with a healthy lifestyle would appreciate the value of lifestyle factors on health more than unhealthy physicians.^10^ Furthermore, physicians with a vulnerability to negative criticism have been shown to feel a greater need to maintain a healthy lifestyle to feel comfortable with the advice they give to patients.^11^

It is also important that patients are willing to follow physicians’ advice. Trust in the physician as a medical expert and the quality of the physician–patient relationship play an essential role in this process.^6,14^ According to an experiment by Frank *et al*, patients have more trust and motivation when a seemingly healthy physician gives advice on eating habits and physical activity compared with physicians who do not disclose healthy lifestyle behaviours.^15^ The research by *Puhl et al* confirms that patients are less likely to follow advice on eating habits from a physician with obesity compared with a physician of healthy weight, regardless of their own body mass index (BMI).^16^ On the other hand, the study by Bleich *et al* shows that patients with a BMI >25 have more trust in an overweight physician’s advice on eating habits but also feel more judged by an overweight physician.^17^ According to Gudzune *et al*, a feeling of being judged harms the physician–patient relationship and consequently also adherence to therapy.^18^ Giving advice empathetically and non-judgementally positively contributes to patient satisfaction, trust in the physician, and their self-reported health.^18–20^

While previous research has shown that a physician’s lifestyle significantly impacts patient adherence to lifestyle advice, the patient’s perspective and self-reported adherence to advice remains largely unexplored. Overall, the literature considering patients’ perspectives currently limits itself to one aspect of physicians’ lifestyle, namely weight. Given that counseling on a healthy lifestyle is an important pillar in both prevention and treatment of chronic diseases, This study aimed to investigate the potential influence of a physician’s lifestyle on a patient’s adherence to advice.

## Method

### Research questions and outcome measures

In this study, we examined the influence of a GP’s unhealthy lifestyle on the adherence to lifestyle advice by the patient. The outcome measure was defined as the degree of willingness to follow the advice.

We examined the need for specific lifestyle advice, including smoking cessation, alcohol use, vaccination, eating habits, physical activity, sleep hygiene and rest, stress and relaxation, and other aspects. The outcome measure was defined as the need for advice on a scale of 0–10. Additionally, we studied whether the GP’s lifestyle influences the feeling of being judged when receiving this advice. The outcome measure was defined as the degree of feeling judged. Finally, we evaluated the influence of a feeling of being judged on the willingness of patients to follow advice. The outcome measure was defined as the degree of willingness to follow the advice.

### Study population

The questionnaire was distributed online via social media (Facebook) and through newsletters of patient organisations. Additionally, we provided information folders with a QR code referring to the survey in the waiting rooms of medical clinics and group practices. The practices were contacted via publicly available email addresses to promote the study.

The questionnaire could be completed from 19 April 2023–12 September 2023, by adults living in Flanders or Brussels with sufficient knowledge of Dutch.

### Study design

The study design is a cross-sectional study without a control group.

We based the survey on the article on core competencies of lifestyle medicine by Lianov *et al* with additional components.^4,5^ The questions were determined after consultation and then presented to a select group of test subjects.

The questionnaire consisted of two parts. In the first part, we inquired about the characteristics of the responders such as gender, age, and subjective physical and mental health on a scale of 0 (very unhealthy) to 10 (very healthy). In the second part, we evaluated the need for specific advice to be rated on a scale of 0 (no need) to 10 (high need). Additionally, we presented statements about the lifestyle of the GP, each highlighting a different unhealthy aspect. Responders had to indicate whether each statement made them more or less likely to follow the advice of this GP compared with a GP with a healthy lifestyle. Subsequently, two more statements were added regarding the feeling of being judged when receiving advice from a GP with an unhealthy lifestyle. Here, responders could indicate whether they felt more or less judged or whether it had no influence. They also had to assess whether the feeling of being judged made them more or less likely to follow the advice or whether it had no influence. Finally, responders had the opportunity to add a free comment.

The survey was offered via Qualtrics KU Leuven.

### Statistical analysis

The data obtained from the above questions were analysed using univariate analyses. Additionally, correlations were calculated using multinomial logistic regressions. Gender, age, subjective physical and mental health, and need for specific lifestyle advice served as independent variables. Adherence to advice, feeling judged, and adherence to advice when feeling judged served as dependent variables. ’No influence’ was always chosen as the reference group. Odds ratios (ORs) with 95% confidence intervals (CIs) were described for each dependent variable in relation to the corresponding independent variables. The significance level was set in advance at 0.05.

These analyses were conducted using SPSS (3.0, 2024). Unanswered questions were treated as ’missing values’.

Finally, we categorised the free comments and linked them to specific concepts based on the Qualitative Analysis Guide of Leuven (QUAGOL) guidelines.^21^

## Results

### Study population

We collected a total of 555 completed questionnaires, of which 466 were included according to the inclusion criteria.

The sample consisted of 342 (73.2%) women and 122 (26.1%) men. Responders had a median age of 26 years, subjective physical health of 8 out of 10, and mental health of 7 out of 10 (Table 1).

**Table 1.** Responder characteristics

Characteristic	% (*n*/*N*)	Median (IQR)
**Gender**		
Woman	73.2 (342/466)	
Man	26.1 (122/466)	
Non-binary or third gender	0.4 (2/466)	
**Age, years**		26 (21.5)
**Subjective physical health**		8/10 (1)
**Subjective mental health**		7/10 (1)

IQR = interquartile range.

### Need for lifestyle advice

The median scores for need of advice on smoking cessation, alcohol use, vaccination, and other aspects ranged from 0–1 out of 10. The median scores for need of advice on eating habits, physical activity, sleep hygiene and rest, and stress and relaxation ranged from 5–6 out of 10 (Table 2).

**Table 2.** Need for lifestyle advice

Need for advice	Median (IQR)
Smoking cessation	0/10 (0)
Alcohol use	0/10 (2)
Vaccination	1/10 (5)
Eating habits	5/10 (5)
Physical activity	5/10 (6)
Sleep hygiene and rest	5/10 (5)
Stress and relaxation	6/10 (5)
Other	0/10 (0)

IQR = interquartile range.

### Influence of GPs’ unhealthy lifestyle on adherence and feeling of judgement

Regarding smoking cessation advice from a GP who smokes, 62.3% (*n* = 291) indicated they were less likely to follow the advice. Regarding advice on alcohol use from a GP with alcohol misuse, 64.9% (*n* = 303) were less likely to follow the advice. For vaccination advice from an unvaccinated GP, 49.7% (*n* = 232) indicated they were less likely to adhere to the advice. For advice on eating habits from an overweight GP, 51.2% (*n* = 239) were less likely to follow the advice. For advice on physical activity from a physically inactive GP, 64.5% (*n* = 301) experienced no influence. Regarding advice on sleep hygiene and rest from a severely tired GP, 62.7% (*n* = 293) indicated it had no influence. For advice on stress and relaxation from a GP with poor work–life balance, 56.3% (*n* = 263) experienced no influence. Regarding receiving advice from a GP with an unhealthy lifestyle, 47.3% (*n* = 220/465) experienced no influence on feeling judged, while 41.1% (*n* = 191) indicated they felt less judged. When feeling judged when receiving advice, 68.8% (*n* = 317/461) indicated they were less likely to follow the advice (Table 3).

**Table 3.** Influence of GPs’ unhealthy lifestyle on adherence and feeling of judgement

Category, % (*n/N*)				Adherence			Feeling of judgement		
				More	Less	No influence	More	Less	No influence
Advice	Smoking cessation	Unhealthy lifestyle of GP	Smoking	5.4 (25/466)	**62.3** (291/466)	32.3 (151/466)	11.6 (54/465)	**41.1** (191/465)	**47.3** (220/465)
	Alcohol use		Alcohol misuse	3.2 (15/466)	**64.9** (303/466)	31.9 (149/466)			
	Vaccination		Unvaccinated	3.9 (18/466)	**49.7** (232/466)	**46.5** (217/466)			
	Eating habits		Overweight	4.3 (20/466)	**51.2** (239/466)	**44.5** (208/466)			
	Physical activity		Physically inactive	2.6 (12/466)	33.0 (154/466)	**64.5** (301/466)			
	Sleep hygiene and rest		Severely tired	9.0 (42/466)	28.3 (132/466)	**62.7** (293/466)			
	Stress and relaxation		Poor work–life balance	9.6 (45/466)	34.0 (159/466)	**56.3** (263/466)			
Feeling of judgement				16.1 (74/461)	**68.8** (317/461)	15.2 (70/461)			

Bold = *P*<0.05.

### Influence of responder characteristics on adherence and feeling of judgement

With increasing age, responders were less likely to choose ’less likely to adhere’ over ’no influence’ regarding adherence when feeling judged (OR 0.973, 95% CI = 0.957 to 0.998) and with advice on smoking cessation (OR 0.979, 95% CI = 0.966 to 0.992), alcohol use (OR 0.983, 95% CI = 0.970 to 0.996), and vaccination (OR 0.975, 95% CI = 0.963 to 0.989) from a GP who violates these domains themselves. Additionally, with increasing subjective physical health, responders were less likely to choose ’less likely to adhere’ over ’no influence’ regarding adherence to advice on physical activity from a physically inactive GP (OR 0.799, 95% CI = 0.677 to 0.942) (Table 4).

**Table 4.** Influence of responder characteristics on less adherence and more feeling of judgement

Category	Less adherence versus no influence, OR (95% CI)								More feeling of judgement versus no influence
	Smoking cessation	Alcohol use	Vaccination	Eating habits	Physical activity	Sleep hygiene and rest	Stress and relaxation	Feeling of judgement	
Gender	0.665 (0.423 to 1.046) *P* = 0.077	1.352 (0.873 to 2.094) *P* = 0.177	1.269 (0.837 to 1.926) *P* = 0.262	0.758 (0.501 to 1.148) *P* = 0.191	1.026 (0.665 to 1.583) *P* = 0.907	0.881 (0.558 to 1.390) *P* = 0.586	1.044 (0.670 to 1.625) *P* = 0.849	1.761 (1.009 to 3.073) *P* = 0.047	0.984 (0.507 to 1.911) *P* = 0.963
Age	**0.979** (0.966 to 0.992) *P* = 0.002	**0.983** (0.970 to 0.996) *P* = 0.008	**0.975** (0.963 to 0.989) *P*<0.001	0.988 (0.976 to 1.001) *P* = 0.066	0.996 (0.983 to 1.009) *P* = 0.545	0.999 (0.985 to 1.013) *P* = 0.880	1.001 (0.988 to 1.015) *P* = 0.847	**0.973** (0.957 to 0.998) *P* = 0.001	1.004 (0.985 to 1.023) *P* = 0.677
Subjective physical health	0.976 (0.829 to 1.149) *P* = 0.768	1.094 (0.930 to 1.287) *P* = 0.277	0.959 (0.821 to 1.121) *P* = 0.602	0.930 (0.795 to 1.089) *P* = 0.367	**0.799** (0.677 to 0.942) *P* = 0.008	0.859 (0.727 to 1.015) *P* = 0.073	0.934 (0.797 to 1.095) *P* = 0.399	0.986 (0.791 to 1.227) *P* = 0.897	0.819 (0.653 to 1.026) *P* = 0.082
Subjective mental health	1.008 (0.866 to 1.174) *P* = 0.918	0.929 (0.797 to 1.083) *P* = 0.345	0.928 (0.803 to 1.071) *P* = 0.307	0.972 (0.844 to 1.119) *P* = 0.689	1.053 (0.908 to 1.220) *P* = 0.494	0.997 (0.854 to 1.164) *P* = 0.972	0.926 (0.797 to 1.075) *P* = 0.313	0.873 (0.704 to 1.082) *P* = 0.214	0.928 (0.747 to 1.152) *P* = 0.496
Need for advice	0.973 (0.900 to 1.053) *P* = 0.502	1.033 (0.948 to 1.124) *P* = 0.459	1.000 (0.940 to 1.063) *P* = 0.997	0.960 (0.900 to 1.024) *P* = 0.213	0.955 (0.893 to 1.021) *P* = 0.175	0.995 (0.928 to 1.066) *P* = 0.879	1.004 (0.939 to 1.075) *P* = 0.899		

Bold = *P*<0.05. OR = odds ratio.

Furthermore, with increasing age, responders were less likely to choose ’feeling less judged’ over ’no influence’ when receiving advice from a GP with an unhealthy lifestyle (OR 0.976, 95% CI = 0.962 to 0.990) (Table 5).

**Table 5.** Influence of responder characteristics on more adherence and less feeling of judgement

Category	More adherence versus no influence, OR (95% CI)								Less feeling of judgement versus no influence
	Smoking cessation	Alcohol use	Vaccination	Eating habits	Physical activity	Sleep hygiene and rest	Stress and relaxation	Feeling of judgement	
Gender	1.095 (0.433 to 2.767) *P* = 0.848	2.623 (0.763 to 9.018) *P* = 0.126	1.795 (0.587 to 5.488) *P* = 0.305	1.109 (0.388 to 3.173) *P* = 0.846	1.573 (0.416 to 5.944) *P* = 0.504	0.703 (0.348 to 1.417) *P* = 0.324	1.456 (0.701 to 3.022) *P* = 0.314	1.395 (0.700 to 2.782) *P* = 0.344	1.029 (0.668 to 1.585) *P* = 0.896
Age	1.012 (0.986 to 1.039) *P* = 0.361	1.013 (0.981 to 1.046) *P* = 0.429	1.025 (0.996 to 1.054) *P* = 0.088	1.010 (0.982 to 1.039) *P* = 0.474	1.022 (0.988 to 1.058) *P* = 0.212	0.994 (0.972 to 1.016) *P* = 0.567	0.998 (0.976 to 1.020) *P* = 0.842	0.987 (0.968 to 1.007) *P* = 0.198	**0.976** (0.962 to 0.990) *P* = 0.001
Subjective physical health	1.170 (0.801 to 1.708) *P* = 0.417	1.329 (0.825 to 2.142) *P* = 0.243	0.804 (0.558 to 1.157) *P* = 0.240	0.894 (0.622 to 1.284) *P* = 0.545	0.913 (0.565 to 1.475) *P* = 0.710	0.890 (0.682 to 1.161) *P* = 0.389	1.044 (0.795 to 1.371) *P* = 0.757	1.094 (0.827 to 1.447) *P* = 0.528	0.965 (0.820 to 1.137) *P* = 0.672
Subjective mental health	0.914 (0.657 to 1.272) *P* = 0.594	0.838 (0.556 to 1.265) *P* = 0.401	1.135 (0.755 to 1.707) *P* = 0.542	1.005 (0.706 to 1.432) *P* = 0.977	1.017 (0.649 to 1.591) *P* = 0.943	1.270 (0.967 to 1.668) *P* = 0.085	1.171 (0.895 to 1.534) *P* = 0.250	0.968 (0.741 to 1.265) *P* = 0.812	1.017 (0.877 to 1.179) *P* = 0.825
Need for advice	1.110 (0.972 to 1.268) *P* = 0.123	1.167 (0.963 to 1.414) *P* = 0.114	0.970 (0.828 to 1.135) *P* = 0.702	1.004 (0.859 to 1.173) *P* = 0.962	1.005 (0.830 to 1.217) *P* = 0.955	1.075 (0.962 to 1.202) *P* = 0.202	1.005 (0.903 to 1.120) *P* = 0.921		

Bold = *P*<0.05. OR = odds ratio.

### Free comments

The most mentioned topic was ’role model function of the GP’ with 11 mentions. This was followed by ’own responsibility for health’ with eight mentions. ’Appreciation for medical advice’ and ’unclearness of questions’ were both mentioned six times. Additionally, ’unknown lifestyle of the GP’, ’limited time and attention during consultations’, and ’understanding approach’ were each mentioned five times. ’Subject-dependent assessment’ was mentioned four times, while ’change of GP’ and ’respect for privacy and professional boundaries’ were each mentioned three times. Finally, ’doubt about the competence and expertise of GPs’ was mentioned two times (Table 6).

**Table 6.** Free comments

Concepts	Mentions, *n*
Role model function of the GP	11
Own responsibility for health	8
Appreciation for medical advice	6
Unclearness of questions	6
Unknown lifestyle of the GP	5
Limited time and attention during consultations	5
Understanding approach	5
Subject-dependent assessment	4
Change of GP	3
Respect for privacy and professional boundaries	3
Doubt about the competence and expertise of GPs	2

## Discussion

### Summary

In this study, we examined the influence of a GP’s unhealthy lifestyle on patient adherence to lifestyle advice. Most responders are less adherent to advice on smoking cessation and alcohol use if their GP respectively smokes or drinks more than average. However, approximately one-third of responders indicated that the GP’s lifestyle does not influence their adherence to these recommendations. The majority, on the other hand, experience no influence when it comes to advice on physical activity, sleep hygiene and rest, and stress and relaxation. Yet, approximately one-third of responders are less adherent when their GP does not apply these recommendations themselves. There is no clear trend regarding vaccination and healthy eating habits. Approximately half of the responders are less likely to follow these recommendations if their GP is not vaccinated or is overweight, respectively.

### Strengths and limitations

This research has some limitations. The hypothetical thinking and formulation of the questions proved to be a challenge. At the same time, working with statements was a strength because it was not necessary to recruit patients from specific physicians with certain unhealthy habits, allowing for a larger population to be surveyed. The major limitation is the limited sample size and the use of a convenience sampling method. We distributed the survey via accessible social media channels, user-group newsletters, and primary care practices. Consequently, we lack insights into response rates and responders’ intentions. However, our study is among the first, to our knowledge, to examine the impact of unhealthy habits of GPs on patient adherence to lifestyle advice.

### Comparison with existing literature

These findings partly contradict previous studies on advice about physical activity and eating habits. Patients have more trust and motivation when a seemingly healthy physician, with a normal BMI, gives advice on physical activity and eating habits compared with an unhealthy physician.^15,16^ On the other hand, some patients with an increased BMI express more trust in an overweight physician’s advice.^17^

The article by O’Brien suggests that some patients prefer a GP without a healthy lifestyle because they feel more understood and less intimidated. Conversely, others prefer a role model as they derive motivation from seeing the end result.^14^ However, we observe that this pattern is not uniform across all themes. It is noteworthy that most responders are less adherent when a GP smokes or drinks more than average. The increasing patient awareness of the harmful effects of smoking and alcohol on health might explain this result.^22^ Most responders experience no influence on the feeling of being judged, while a large portion feel less judged when receiving advice from a GP without a healthy lifestyle. These findings do not align with the research by Bleich *et al*, which found that patients with a BMI >25 feel more judged by an overweight GP.^17^ Moreover, a clear majority are less adherent when they feel judged. This aligns with existing literature. A feeling of being judged harms the physician–patient relationship and consequently adherence to therapy.^18–20^

Age weakly predicts the feeling of being judged and adherence. This is only true for adherence when feeling judged and advice on smoking cessation, alcohol use, and vaccination from a GP who violates these domains. Older responders experience less influence from an unhealthy lifestyle of GPs when compared with younger responders. This may stem from their upbringing in a time of medical paternalism when physicians were seen as infallible authorities and their advice was often blindly followed.^23^ Moreover, the general social acceptance of smoking and alcohol use in the past could explain why this mainly applies to these specific behaviour patterns.^24^ Vaccination programmes also played a less prominent role in health care at the time.^25^

Finally, several themes emerged in the free comments. Various opinions were expressed regarding expectations about the lifestyle of GPs. A common opinion is that GPs should serve as role models, with some responders even considering changing their GP if this is not the case. Conversely, responders indicate that they hold themselves responsible for their health but appreciate any medical advice, especially if given in an understanding manner. They respect the privacy and professional boundaries of GPs. Responders also feel that during consultations, limited time and attention is often devoted to lifestyle and they express doubts about the competence and expertise of GPs in lifestyle medicine. According to the literature, many physicians also acknowledge the lack of time and training as significant barriers.^6,7,26^ Overall, it is crucial to promote a healthy lifestyle among physicians themselves, for both their own wellbeing and that of their patients.^14,15^

### Implications for research and practice

By focusing on a diverse sample from Flanders and Brussels, the findings underscore the importance of GPs modeling healthy behaviours to enhance patient trust and adherence. These insights are crucial for developing strategies to improve patient outcomes in lifestyle-related chronic disease prevention and management. The study’s results emphasise the need for targeted interventions to support GPs in maintaining healthy lifestyles.

In the future, it would be beneficial to further explore ways to promote and emphasise the exemplary role of medical doctors in enhancing patients’ adherence to lifestyle advice.

In conclusion, this research shows that the influence of a GP’s unhealthy lifestyle on patient adherence to lifestyle advice is not unequivocal. Most responders indicate that they are less adherent to advice on smoking cessation and alcohol use when their GP respectively smokes or drinks more than average, while the majority report no influence of the GP’s lifestyle on other types of lifestyle advice. Nevertheless, approximately one-third of the responders are less adherent when their GP does not follow these recommendations themselves. Therefore, it remains essential to promote a healthy lifestyle among GPs. Furthermore, responders are less adherent when they feel judged by their GP. Therefore, it is important that advice is given in an empathetic and non-judgemental manner.

## Data

The dataset relied on in this article is available from the first author on reasonable request.
